# Supplementary material for: Public engagement for inclusive and sustainable governance of climate interventions
Source: Nat Commun. 2024 May 16;15:4168. doi: 10.1038/s41467-024-48510-y (PMC11099155; doi:10.1038/s41467-024-48510-y)
Supplement: Supplementary file 1 — Supplementary Information [file 41467_2024_48510_MOESM1_ESM.pdf]

SUPPLEMENTARY INFORMATION

Public engagement for inclusive and sustainable governance of climate interventions

Livia Fritz [1*], Chad M. Baum[1], Sean Low[1], Benjamin K. Sovacool[1-3]

[1] Department of Business Development and Technology, Aarhus University, Birk Centerpark 15, 7400 Herning, Denmark
[2] Science Policy Research Unit (SPRU), University of Sussex Business School, Jubilee Building, Arts Rd, Falmer, Brighton BN1 9SL, United Kingdom
[3] Department of Earth and Environment, Boston University, 685 Commonwealth Ave, Boston, MA 02215, United States

* Correspondence and requests for materials should be addressed to Livia Fritz (email: livia.fritz@btech.au.dk)

A. SUPPLEMENTARY METHODS

Project and mixed methods framework. Our study is part of project Geoengineering and Negative Emissions Pathways in Europe (GENIE), a multi-institutional assessment of carbon removal and solar geoengineering approaches. The following research framework forms the basis for several emerging publications and other contributions. We utilize a multi-methods framework combining a survey instrument with focus groups.

The focus groups' design is described below. We foreground that focus groups – reflecting small sample sizes, screening for particular characteristics, and potentially driven by emergent topics and dynamics particular to each group – should not be seen as (wholly) representative of a national public. Here, we aim at the in-depth treatment and detail that comes with deliberation, and comparison with the wider public perceptions literature. Furthermore, focus groups frequently refer to their local or national contexts – which is significant and can form the basis for further situated assessment, even if not definitively representative.

Participation and recruitment. 2 focus groups – one each for urban and rural settings – were held in each of the 22 countries, for a total of 44 focus groups. Recruitment aimed at 8 participants per focus group. Due to technical difficulties and dropouts, the lowest number of participants was 5. The total number of participants was 323. Recruitment was conducted in collaboration with Norstat, a European-based data collection company (https://norstatgroup.com/). The following financial compensation was provided to each participant:

- UK £50
- Germany Euro 55
- Spain Euro 40
- USA $75
- Indonesia 70800 IDR
- South Africa R1000
- Kenya KES840
- Nigeria NGN 46000
- China average RMB 400
- Brazil 360 Real
- KSA SAR250
- Turkey 1500 Lira
- Norway 800 Norwegian Krona
- Sweden 800 Swedish Krona
- India INR 9500
- Italy Euro 50
- DomRep RD$ 1300
- Poland PLN 160
- Austria Euro 80
- Switzerland Euro 100
- Australia $150 AUD
- Chile US $45

Prospective participants were screened via an online survey for a number of mandatory and 'soft' criteria. *Climate denialism* was screened out (all who answered "No: "Do you believe climate change is happening?"). Focus groups were further screened for an even split between *genders*, and between *young (18-44) and old (above 45+) cohorts*. Participants were screened for an urban (including suburban) or rural background, which was self-defined, and relied on responding "Urban", "Suburban", or "Rural" to the question: "How would you describe the area in which you live?".

Two further 'soft' – guiding but not mandatory – screens were held. The first was for distribution across *education level, income, and occupation type*, each tailored by country. The second was for distribution across *regions within a country*. In most cases, these were defined by formal (e.g federal) administrative regions or states/provinces; in a smaller number, these were defined by broad geographic regions (in USA, India, Brazil, Indonesia).

Materials. Two sets of materials – a *discussion guide* (of questions, topical emphases, and timings) for moderators, and *information materials on approaches* (distributed to public participants beforehand) – were developed primarily by the authors, in collaboration with Norstat. Materials were written originally in English and communicated in that language with focus groups in US, UK, Kenya, Nigeria, South Africa, Australia, and India (urban). Materials for other countries were translated into: German (Germany, Austria, Switzerland); Italian (Italy); Polish (Poland); Norwegian (Norway); Swedish (Sweden); Spanish (Spain, Chile, Dominican Republic); Portuguese (Brazil); Mandarin Chinese (China); Turkish (Turkey); Arabic (Saudi Arabia), and Bahasa Indonesia (Indonesia), Hindi (India rural). All technical terms were translated from English into their native languages by academic experts (all colleagues in climate and energy governance known to the authors).

Discussion guide. This consisted of the following themes in four groupings:

Hopes (prospective benefits)

1. What are the benefits from any of these approaches?
2. Who might gain the most from these benefits, and why?

- Alternative way to tackle if needed - If these were implemented in your community/country, who would be affected (how? why?)

Concerns (prospective risks)

3. What are the risks from any of these approaches?
4. Who might be most negatively impacted from these risks, and why?

- Alternative way to tackle if needed - If these were implemented in your community/country, who would be affected (how? why?)

Corresponding governance

5. In an ideal world who are the most significant people that should help make decisions on this approach – in your community, or your country, or even the world?
6. What actions should be taken before there is consideration to implement this approach ... what would you like to see done?
7. How would you want yourself, and the wider public, to be involved in making decisions on these approaches?

Headlines exercise

8. A creative mini-scenario exercise was held, in which participants were asked to create a (newspaper) headline in 2030, with four elements: an approach, an actor, and an event, in sum representing a good or bad outcome related to the approach (a headline that makes the participant feel hopeful or worried).

The guiding logic was to focus conversation on actors, actions, and agendas at the most tangible scale possible.

Informational materials on approaches. This was sent to participants a week prior to the conduct of the focus group. Participants were encouraged to do further research, and to discuss with family, friends, and members of their community – this was a step directly taken from the engagement process of Cox et al., 2020a, and other publications of that research group. The information materials consisted of the following elements. There was one introductory page each for carbon removal and solar geoengineering as separate suites, with bullet points on their overarching characteristics. The carbon removal introductory page emphasized a spectrum between biogenic (afforestation and reforestation; soil carbon sequestration), to chemical /

engineered (DACCS, enhanced weathering), to a mix thereof (BECCS). The materials were careful not to use the term “nature-based”, understanding that the term has documented steering effects (Corner et al., 2013; Osaka et al., 2021). Instead, materials referred to these as approaches “that change how we use nature”. Chemical / engineered systems were referred to as utilizing “large-scale engineering systems”. In following pages, each approach was accompanied by a column of approach-specific text, taking up one-third to half a page each. Each column contained: a brief technical description; a ‘cartoon’ picture, deliberately stylized to avoid reification; a short list of technical infrastructural needs; and a point or two each of key technical pros and cons that were extremely abbreviated to forestall as much framing as possible. We are grateful for and acknowledge the assistance of William Lamb and the Mercator Research Institute on Global Commons and Climate Change (MCC) with helping to design and providing funding for the graphics for each of the technologies.

We include these materials below:

- During our focus group in a few days, we will be discussing a number of **new ideas for helping to address climate change**.
- This document contains **information** about these new ideas. **Please read it carefully, as it will help our discussions!**
- We **do not** expect you to have any prior or special knowledge - most do not.
- We are interested in your reactions to these ideas, and the **benefits and concerns** that you think they may bring to **yourself, your community, your country, or the world.**
- We understand that there are many approaches. You don’t have to learn everything by heart – please consider beforehand which approaches you find **most interesting**, or **most worrying**. We encourage you to **discuss them with family and friends** before the focus group.

CLIMATE CHANGE

The **Earth's temperature is increasing** as a result of the human-driven release of **carbon dioxide (CO2)** and other greenhouse gases from the **burning of fossil fuels** (e.g. oil, gas, coal).

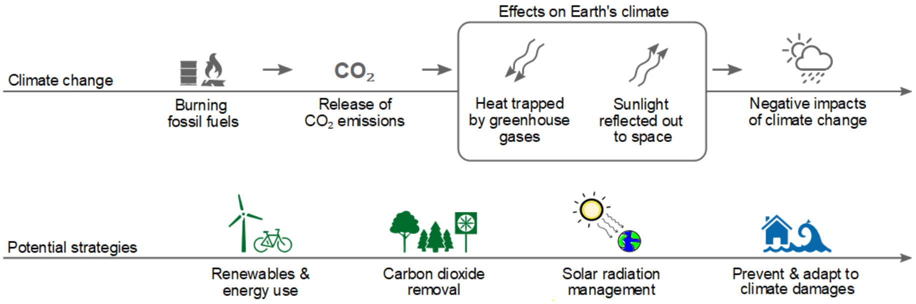

Potential strategies to help limit the effects of climate change include reducing carbon emissions by **using renewable energy** or **preventing and adapting to climate damages**.

Together, we will discuss two new suggestions for helping address climate change:

SOLAR RADIATION MANAGEMENT and **CARBON DIOXIDE REMOVAL**

2

SOLAR RADIATION MANAGEMENT

reflects some sunlight back into space, cooling the planet, and reducing climate change impacts.

- **None of these ideas are used or proven in the real world.**
- If they work, these approaches would **cool temperatures quickly** (weeks to months).
- They might lessen some effects of climate change, but **would not reduce our carbon emissions**.
- Furthermore, if we do not reduce carbon emissions while reflecting sunlight to cool temperatures, and **we stopped reflecting sunlight, temperatures would rise again**.

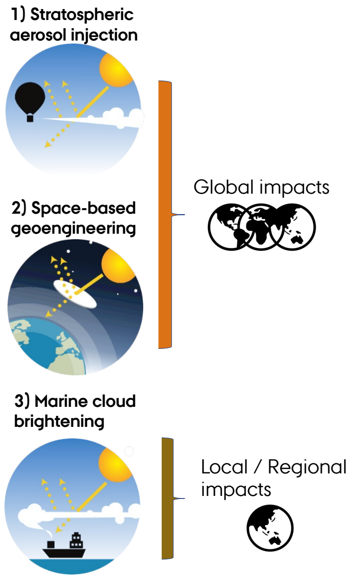

3

If used, these approaches would have global effects, and cannot be limited to a single country.

1) Stratospheric Aerosol Injection

would use aircraft to spray small reflective particles into the upper atmosphere. Air currents would carry the particles around the planet.

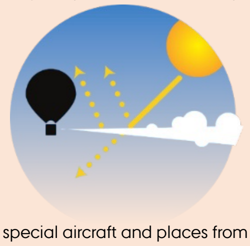

Needs special aircraft and places from where they can be launched.

- Temperatures would cool globally, reducing many impacts of climate change.

- Cooling of temperatures would not be equal in all countries or regions.
- Effects (e.g. on rainfall and crops) would vary.
- Knowledge about side-effects is limited.
- One wealthy country alone could deploy this technology.

2) Space-based Geoengineering

would put a giant "mirror" of reflective material in outer space between the Earth and the sun.

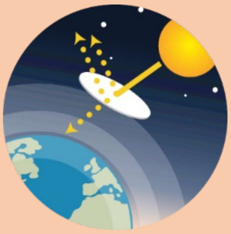

Needs space-craft and advanced materials.

- Temperatures would cool globally, reducing many impacts of climate change.

- Cooling of temperatures might not be equal in all countries or regions.
- Effects (e.g. on rainfall and crops) would vary.
- Knowledge about side-effects is limited.

… regional effects, and can be limited to a smaller area.

3) Marine cloud brightening

would spray small particles, such as sea salt, into the air over the oceans, to make clouds brighter.

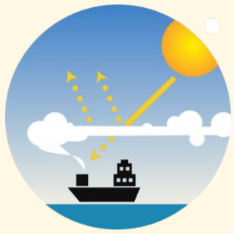

Needs specially designed ships made to spray the particles.

- Temperatures would cool locally or regionally.
- May protect marine or coastal ecosystems threatened by climate change.

- More effective over marine areas than land.
- Effects are unpredictable beyond where it is locally used.

4

CARBON DIOXIDE REMOVAL

absorbs CO2 from the air – using new technologies or changing how we grow trees and crops.

- Most of these approaches have **been proven in the real world at small scales** (the size of farms or factories).
- At large scales, we do not know their full costs and side-effects.
- If they work, these approaches would **reduce temperatures slowly** (over decades).

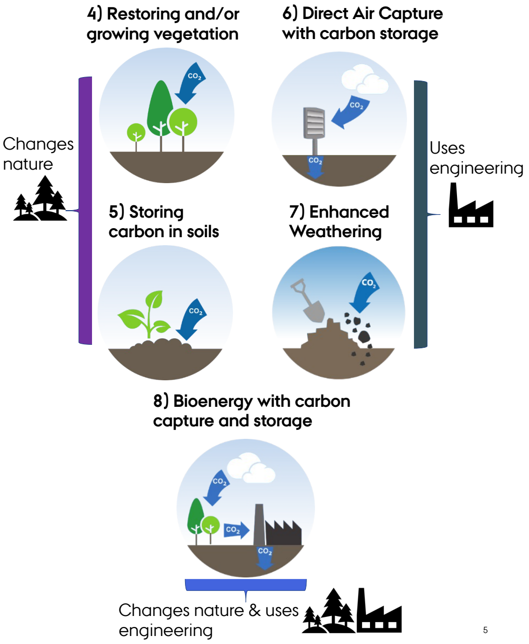

5

These approaches would change how we use nature (forests, marine areas, and soils).

4) Restoring and/or growing vegetation: One kind of approach **restores vegetation where it had once grown well.** Examples: restoring trees, or mangroves and coastal wetlands. Another kind **grows vegetation in new areas**. Examples: growing trees, or seaweed plantations.

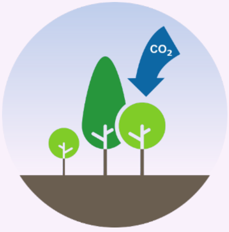

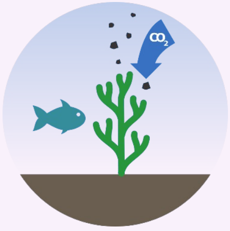

Needs new land and marine areas, or changes to existing areas from how they are presently used.

- Could improve livelihoods in rural farming or coastal communities (By farming & growing vegetation in the same area; by defending against soil erosion or floods).
- Could compete with livelihoods (By using space for growing vegetation, instead of for crops).
- If the vegetation (forest, mangroves) is destroyed, the carbon will go back into the air.

5) Storing carbon in soils: These approaches increase how much carbon is stored in soils, by changing agricultural practices and adding (new kinds of) plant materials. **Soil carbon sequestration** rotates crops, puts crop residues on the field (e.g. husks, leaves), and/or combines the use of land for trees, crops and agriculture (agro-forestry). **Biochar** is a charcoal-like substance that is made by heating up forestry and agricultural waste. Adding this substance to soil, carbon can be stored.

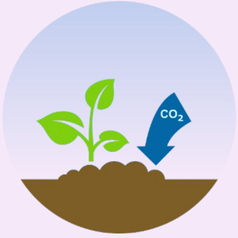

Needs: Soil carbon sequestration needs agricultural lands. **Biochar** needs energy and agricultural lands.

- Both **soil carbon sequestration** and **biochar** could improve soils for farming.
- **Biochar** is a material that could also help make some industrial products (e.g. concrete) and farming products (e.g. animal feed).
- The plant materials used for making **biochar** could serve other purposes.

These approaches depend on large-scale engineering and chemical systems.

6) Direct Air Capture with Carbon Storage uses large fans and filters to remove CO_2 from the air, and then turn the CO_2 to a liquid that can be stored underground.

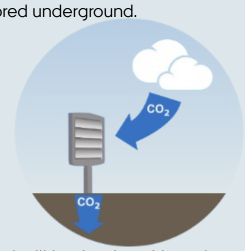

Needs facilities the size of factories, energy, pipelines to transport carbon, and underground places to store carbon.

- Captured CO_2 could be used to make some products (e.g. airplane fuels, building materials).
- High energy needs: Should this be provided by renewables or fossil fuels?
- Oil companies could pay for captured CO_2 to drive out more fossil fuels from existing wells.
- Carbon could leak, depending on how and where it is transported and stored.

7) Enhanced Weathering grinds different kinds of rocks, before placing them onto farmlands, beaches, or next to rivers. Normally, such rocks absorb CO_2 as they very slowly erode. Enhanced weathering speeds up this process.

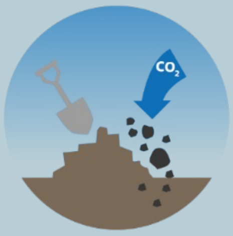

Needs mining and transport of different rocks (limestone, basalt), and energy for grinding rock.

- Some kinds of rock could improve soils for farming.
- Could use waste products from the mining industry instead of mining new rocks.
- Increased mining and energy use.

… large-scale engineering AND change how we use nature.

8) Bioenergy with Carbon Capture and Storage involves growing plants or crops that absorb CO_2 from the air. It then burns those plants or crops for energy and uses technology to capture the CO_2 released, storing the carbon underground.

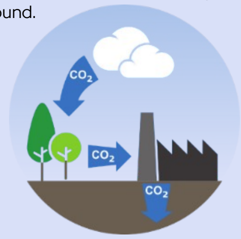

Needs plants and crops, land, and water, and underground places to store carbon.

- Bioenergy could power homes and industries.
- Growing crops for bio-energy could compete with growing crops for food.
- Carbon could leak, depending on how and where it is transported and stored.

Moderation. Meetings in the majority of countries were conducted online. Meetings in the Dominican Republic, Nigeria, Kenya, South Africa and India rural were held in person or in hybrid format. They were moderated by Norstat personnel, in the same language in which materials were translated into (see Materials for the list of native languages). All focus groups ran for at least 2 hours, with the carbon removal suites receiving 10 more minutes than the solar geoengineering suites. Half of the focus groups began by discussing carbon removal, and the other half with solar geoengineering.

Transcription. Each focus group was recorded in its entirety, and transcribed by Norstat. All transcripts went through multiple rounds of clarification between the transcribers and the authors to ensure accuracy and quality.

B. SUPPLEMENTARY TABLES AND FIGURES

Supplementary Figure 1. Relative importance of forms of engagement in the 22 countries, according to number of coded segments
Note: symbol size refers to column, the bigger the square, the greater the number of coded segments for the respective engagement format

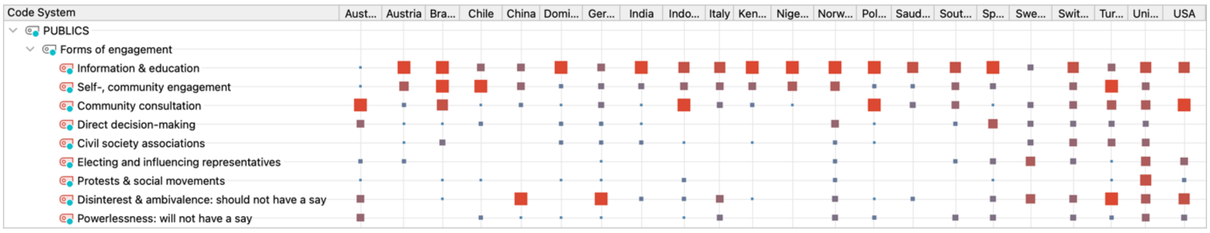

Supplementary Figure 2. Relative importance of forms of engagement with the respective CDR and SRM approaches, according to number of coded segments
Note: symbol size refers to row, the bigger the square, the greater the number of coded segments for the respective engagement format

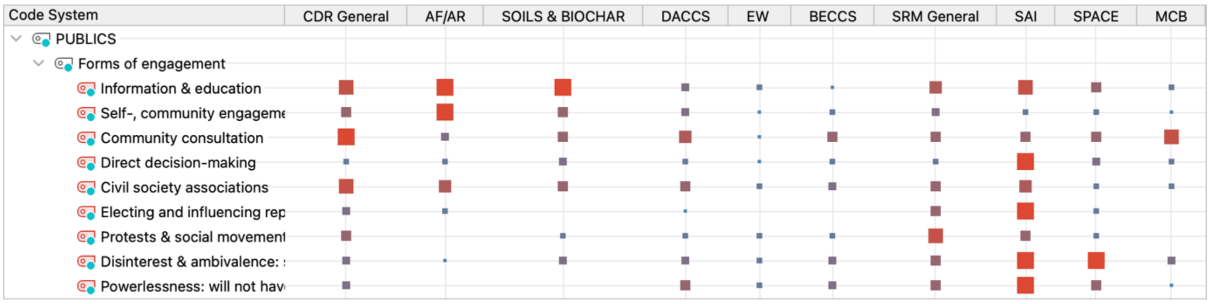

Supplementary Figure 3. Relative importance of rationales for/against engagement in the 22 countries, according to number of coded segments
Note: symbol size refers to row, the bigger the square, the greater the number of coded segments for the respective rationale and approach

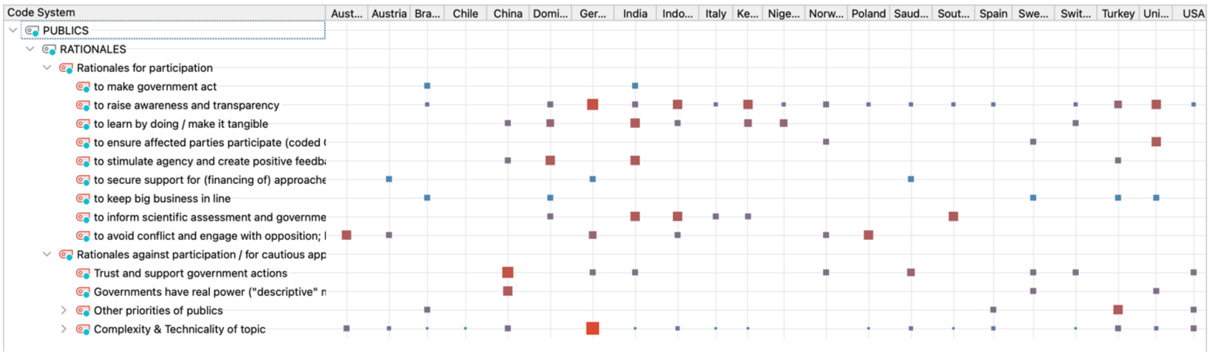

Supplementary Figure 4. Relative importance of rationales for/against public engagement with the respective CDR and SRM approaches, according to number of coded segments
Note: symbol size refers to row, the bigger the square, the greater the number of coded segments for the respective rationale and approach

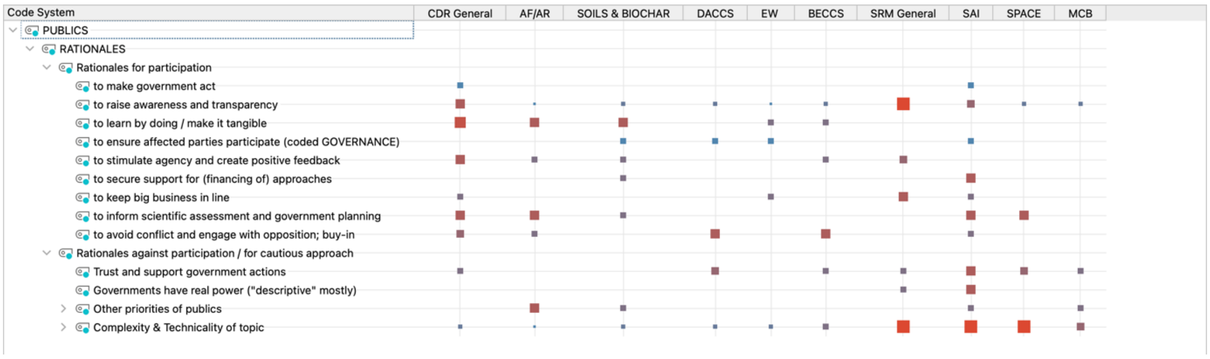
Code System
CDR General
AF/AR
SOILS & BIOCHAR
DACCS
EW
BECCS
SRM General
SAI
SPACE
MCB
PUBLICS
RATIONALES
Rationales for participation
to make government act
to raise awareness and transparency
to learn by doing / make it tangible
to ensure affected parties participate (coded GOVERNANCE)
to stimulate agency and create positive feedback
to secure support for (financing of) approaches
to keep big business in line
to inform scientific assessment and government planning
to avoid conflict and engage with opposition; buy-in
Rationales against participation / for cautious approach
Trust and support government actions
Governments have real power ("descriptive" mostly)
Other priorities of publics
Complexity & Technicality of topic
