# Supplementary material for: Public engagement for inclusive and sustainable governance of climate interventions
Source: Nat Commun. 2024 May 16;15:4168. doi: 10.1038/s41467-024-48510-y (PMC11099155; doi:10.1038/s41467-024-48510-y)
Supplement: Supplementary file 3 — Reporting Summary [file 41467_2024_48510_MOESM3_ESM.pdf]

nature portfolio

Corresponding author(s): Livia Fritz

Last updated by author(s): Apr 8, 2024

Reporting Summary

Nature Portfolio wishes to improve the reproducibility of the work that we publish. This form provides structure for consistency and transparency in reporting. For further information on Nature Portfolio policies, see our Editorial Policies and the Editorial Policy Checklist.

Statistics

For all statistical analyses, confirm that the following items are present in the figure legend, table legend, main text, or Methods section.

n/a	Confirmed	
☒	☐	The exact sample size (*n*) for each experimental group/condition, given as a discrete number and unit of measurement
☒	☐	A statement on whether measurements were taken from distinct samples or whether the same sample was measured repeatedly
☒	☐	The statistical test(s) used AND whether they are one- or two-sided<br>*Only common tests should be described solely by name; describe more complex techniques in the Methods section.*
☒	☐	A description of all covariates tested
☒	☐	A description of any assumptions or corrections, such as tests of normality and adjustment for multiple comparisons
☒	☐	A full description of the statistical parameters including central tendency (e.g. means) or other basic estimates (e.g. regression coefficient) AND variation (e.g. standard deviation) or associated estimates of uncertainty (e.g. confidence intervals)
☒	☐	For null hypothesis testing, the test statistic (e.g. *F*, *t*, *r*) with confidence intervals, effect sizes, degrees of freedom and *P* value noted<br>*Give P values as exact values whenever suitable.*
☒	☐	For Bayesian analysis, information on the choice of priors and Markov chain Monte Carlo settings
☒	☐	For hierarchical and complex designs, identification of the appropriate level for tests and full reporting of outcomes
☒	☐	Estimates of effect sizes (e.g. Cohen's *d*, Pearson's *r*), indicating how they were calculated

Our web collection on statistics for biologists contains articles on many of the points above.

Software and code

Policy information about availability of computer code

Data collection	No such software was used.
Data analysis	Analysis was conducted using MaxQDA software, a software facilitating qualitative data analysis.

For manuscripts utilizing custom algorithms or software that are central to the research but not yet described in published literature, software must be made available to editors and reviewers. We strongly encourage code deposition in a community repository (e.g. GitHub). See the Nature Portfolio guidelines for submitting code & software for further information.

Data

Policy information about availability of data

All manuscripts must include a data availability statement. This statement should provide the following information, where applicable:

- Accession codes, unique identifiers, or web links for publicly available datasets
- A description of any restrictions on data availability
- For clinical datasets or third party data, please ensure that the statement adheres to our policy

The dataset generated and analyzed during the current study are not publicly available at present, to allow for further analysis and publication of findings over the course of the project. Access to the raw data can be made available from the corresponding author on reasonable request. The totality of the dataset will be made publicly available in full following the conclusion of the GENIE project.

Research involving human participants, their data, or biological material

Policy information about studies with human participants or human data. See also policy information about sex, gender (identity/presentation), and sexual orientation and race, ethnicity and racism.

Reporting on sex and gender	Sex and gender are not considered as part of the analysis of this paper.
Reporting on race, ethnicity, or other socially relevant groupings	Race and ethnicity were not considered as part of the analysis of this paper.
Population characteristics	Focus groups were screened for an even split between genders, between 18-44 yrs and 45+ yrs cohorts, division between urban and rural, and for distribution across education level, income, occupation type, and region.
Recruitment	Recruitment was conducted in collaboration with Norstat via an online survey for characteristics in 'population', above
Ethics oversight	All components of the research were granted ethical approval by relevant authorities at Aarhus University (#2021-13)

Note that full information on the approval of the study protocol must also be provided in the manuscript.

Field-specific reporting

Please select the one below that is the best fit for your research. If you are not sure, read the appropriate sections before making your selection.

☐ Life sciences ☒ Behavioural & social sciences ☐ Ecological, evolutionary & environmental sciences

For a reference copy of the document with all sections, see nature.com/documents/nr-reporting-summary-flat.pdf

Behavioural & social sciences study design

All studies must disclose on these points even when the disclosure is negative.

Study description	Qualitative study deploying 44 focus groups in 22 countries to gauge public perceptions on carbon removal technologies
Research sample	44 focus groups, each with 5-8 participants, totalling 323. Screening criteria detailed in sampling strategy, below.
Sampling strategy	Focus groups were screened for an even split between genders, between 18-44 yrs and 45+ yrs cohorts, division between urban and rural, and for distribution across education level, income, occupation type, and region.
Data collection	Data was collected by recording over video conferencing softwares such as Zoom, and transcribed and translated by Norstat.
Timing	Data collection proceeded from October to December 2022.
Data exclusions	No data was excluded from the current study.
Non-participation	Participants were told, at the stage of providing full and informed consent before the beginning of the study, and that they had the right to withdraw participation at any time. All identities have been anonymized, at every stage of transcription and analysis.
Randomization	This condition does not apply to our methodology.

Reporting for specific materials, systems and methods

We require information from authors about some types of materials, experimental systems and methods used in many studies. Here, indicate whether each material, system or method listed is relevant to your study. If you are not sure if a list item applies to your research, read the appropriate section before selecting a response.

Materials & experimental systems

n/a	Involved in the study	
☒	☐	Antibodies
☒	☐	Eukaryotic cell lines
☒	☐	Palaeontology and archaeology
☒	☐	Animals and other organisms
☒	☐	Clinical data
☒	☐	Dual use research of concern
☒	☐	Plants

Methods

n/a	Involved in the study	
☒	☐	ChIP-seq
☒	☐	Flow cytometry
☒	☐	MRI-based neuroimaging

Plants

Seed stocks	*Report on the source of all seed stocks or other plant material used. If applicable, state the seed stock centre and catalogue number. If plant specimens were collected from the field, describe the collection location, date and sampling procedures.*
Novel plant genotypes	*Describe the methods by which all novel plant genotypes were produced. This includes those generated by transgenic approaches, gene editing, chemical/radiation-based mutagenesis and hybridization. For transgenic lines, describe the transformation method, the number of independent lines analyzed and the generation upon which experiments were performed. For gene-edited lines, describe the editor used, the endogenous sequence targeted for editing, the targeting guide RNA sequence (if applicable) and how the editor was applied.*
Authentication	*Describe any authentication procedures for each seed stock used or novel genotype generated. Describe any experiments used to assess the effect of a mutation and, where applicable, how potential secondary effects (e.g. second site T-DNA insertions, mosiacism, off-target gene editing) were examined.*
